# Supplementary material for: Significant changes in the skin microbiome mediated by the sport of roller derby
Source: PeerJ. 2013 Mar 12;1:e53. doi: 10.7717/peerj.53 (PMC3628844; doi:10.7717/peerj.53)
Supplement: Supplemental Information 1 [file peerj-01-53-s001.docx]

**Supplemental Primer/Adapter Sequences:**

Illumina adapters were included only partially during the first PCR, and extended during a second PCR using the sequences below.

Adapter + primer sequences for PCR1:

5’ TCTCGGCATTCCTGCTGAACCGCTCTTCGATCT-XXXXXX- GTGCCAGCMGCCGCGGTAA 3’ 5’ ACACTCTTTCCCTACACGACGCTCTTCCGATCT-XXXXXX- TACNVGGGTATCTAATCC 3’

where XXXXXX = 6bp barcode added to each primer

Adapter addition sequences for PCR2:

5’ AAGCAGAAGACGGCATACGAGATCGGTCTGGCATTCCTGC 3’

5’ ATGATACGGCGACCACCGAGATCTACACTCTTTCCCTACACGACG 3’.
